# Efficacy of a Novel Intraoperative Surgical Irrigant in Preventing Periprosthetic Joint Infections in Primary Knee, Hip, and Shoulder Arthroplasties: A Retrospective Analysis

**DOI:** 10.1111/os.14052

**Published:** 2024-04-16

**Authors:** Marshall Williams, Robert M. Harris

**Affiliations:** ^1^ Jack Hughston Memorial Hospital Phenix City AL USA; ^2^ Hughston Foundation Columbus GA USA; ^3^ Quillen College of Medicine, East Tennessee State University Johnson City TN USA

**Keywords:** Biofilm, Intraoperative irrigant, Periprosthetic joint infection, Primary joint arthroplasty, Retrospective study

## Abstract

**Objective:**

Primary joint arthroplasty (JA) is one of the most common operating room (OR) procedures, with knee and hip arthroplasties being listed in the top five most frequent OR procedures and while not as common, shoulder arthroplasties are increasing at greater rates than knee and hip arthroplasties. Periprosthetic joint/shoulder infections (PJI/PSI) are a devastating complication of primary JAs with infection prevention deemed as the single most important strategy in combating them. The objective of this study was to retrospectively evaluate the efficacy of XPERIENCE® Advanced Surgical Irrigation (XP) in preventing PJI following primary joint arthroplasty.

**Methods:**

This is a retrospective study of primary knee, hip and shoulder arthroplasties that were performed by multiple orthopedic surgeons at a single hospital setting. XPERIENCE was used as an intraoperative surgical irrigant either solely, or with other intraoperative practices for prevention of infection. Incidence of acute PJI occurring within 90 days of index surgery were retrospectively collated.

**Results:**

Four hundred and twenty‐three (423) primary joint replacement surgeries treated intraoperatively with XP, were evaluated for acute PJI incidence. Retrospective evaluations determined that 95% of the subjects had at least one risk factor predisposing them to PJI. There were zero PJIs diagnosed in the knee and hip arthroplasty cohorts and zero PSIs diagnosed in the shoulder arthroplasty cohorts.

**Conclusion:**

The absence of PJI/PSI diagnoses in the JA cohorts treated intraoperatively with XP indicates that it could be an efficacious antimicrobial irrigant in preventing PJI, and warrants being evaluated in prospective, randomized controlled clinical trials as the sole intraoperative irrigant, as well as in combination with the other intraoperative infection prevention regimens evaluated in this retrospective study.

## Graphical abstract

This is a retrospective study of 423 primary joint arthroplasties that were performed by multiple orthopedic surgeons at a single hospital setting. A unique intraoperative surgical irrigant was used which resulted in zero incidences of periprosthetic joint infection within 90 days post index surgery.

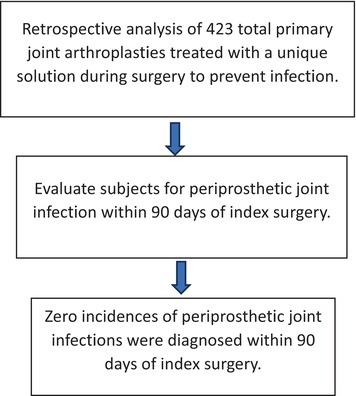

## Introduction

Primary hip and knee arthroplasties are two of the most common elective surgical operating room (OR) procedures performed in the USA.^1^ A projected analysis using the 2014 US National Inpatient Samples (NIS) database determined that the annual number of total hip arthroplasties (THA) are predicted to increase by 129% while the annual number of total knee arthroplasties (TKA) are predicted to increase by 182% by 2030.^2^ Similar predictive modeling for total shoulder arthroplasties (TSA) using 2017 US NIS values, estimated an increase of 235% by 2025.^3^ Alongside the substantial increases estimated in joint arthroplasty (JA), concurrent increases in devastating complications like PJI/PSI, defined as infections involving the joint prosthesis and adjacent tissue, can cause significant morbidity, mortality and added costs to health systems and service payers and is one of the more common causes for revision associated with failed joint replacement. Combined annual hospital costs related to PJI of the hip and knee are predicted to increase to $1.85 billion by 2030 while PSI costs are predicted to reach nearly $500 million annually by 2030 in the USA.^4^, ^5^ Infection prevention is deemed the most important strategy to combat SSI/PJI.^6^, ^7^

Organizations like the Centers for Disease Control and Prevention (CDC), the American Academy of Orthopedic Surgeons (AAOS) and Musculoskeletal Infection Society (MSIS) have proposed clinical practice guidelines for the definition, diagnosis, and management of PJI/PSI.^8^, ^9^, ^10^, ^11^ Depending on the time of onset, PJI can be subdivided into three stages: (i) early PJI occurring ≤90 days from index surgery; (ii) delayed PJI occurring between 3 and 12 months from index surgery; and (iii) late PJI occurring >12 months from index surgery.^12^ The overall incidence of PJI in hip and knee arthroplasty can occur at greater than 2%^4^, ^11^, ^12^, ^13^, ^14^ while reported rates of PSI following shoulder arthroplasty can range from 0.4% to 4%.^15^, ^16^

The National Institute of Health (NIH) estimates that 80% of microbial infections in the body are attributable to the formation of biofilms.^17^ Biofilms are complex microbial communities sheathed within a protective matrix of self‐produced extracellular polymeric substances (EPS) composed of polysaccharides, proteins, glycoproteins, glycolipids, and extracellular DNA.^18^ The formation of biofilms is intrinsic to the pathogenesis of PJI.^19^, ^20^, ^21^, ^22^, ^23^ Exogenously introduced or endogenously present contaminating microbial bioburden can colonize the surgical wound and prosthesis during intraoperative procedures. Once attached to the surgical wound host tissue and/or prosthetic material, microbial biofilm structures can form rapidly within a few hours and mature to exhibit various defense mechanisms like recalcitrance to antimicrobial treatments and host immune defenses as well as cause skewing of host immune defenses making the eradication of the biofilm particularly difficult.^24^, ^25^

Preventing the microbial contamination and colonization of the surgical wound space is identified as the most important strategy in combating PJI.^6^ The use of multi‐tiered perioperative strategies to decrease contaminating microbial bioburden, is recommended to prevent the formation of biofilm and subsequent PJI, with intraoperative irrigation of the surgical space using antiseptic or antimicrobial solutions determined to be an accepted infection prevention practice.^26^, ^27^, ^28^, ^29^ However, there is no universally accepted standard of care (SOC) practice for intraoperative surgical irrigation.

In summary, with the substantial increase in the number of elective JA surgeries, there is a concomitant increase in the number of biofilm‐associated PJI/PSI. This places a high economic burden on the healthcare industry, but more importantly, significantly increases morbidity impacting patients' quality of life and increases mortality. Decreasing the number and incidence rate of PJI/PSI is imperative. This retrospective study was designed in a real‐world hospital setting with multiple surgeons conducting their preferred intraoperative infection prevention protocols in subjects undergoing primary knee, hip, or shoulder arthroplasty. All surgeons utilized the novel intraoperative surgical irrigant, XPERIENCE® Advanced Surgical Irrigation (XP) which was developed based on a technology platform to prevent and treat biofilm.^30^ The primary aim of the study was to determine acute PJI/PSI incidence rate in the XP treated cohorts for comparison to historically reported incidence rates in the peer reviewed literature. The secondary aims were to evaluate the mean length of hospital stay and mean length of surgery to evaluate any correlation with infection.

## Methods

### Procedures

Primary knee, hip and shoulder arthroplasty procedures were performed between June 2021 and June 2022 at Jack Hughston Memorial Hospital in Phenix City, Alabama. The surgeries were performed by nine different orthopedic surgeons using their own preferred state of the art techniques, surgical approaches, infection prevention routines and other SOC procedures. XPERIENCE® Advanced Surgical Irrigation (Next Science® Jacksonville, FL, USA), was used intraoperatively and flushed into the surgical wound space *via* pulsed or manual lavage for cleansing of the surgical wound space, during various stages of the arthroplasty procedure and/or at the end of the procedure, just prior to surgical wound closure, depending on the surgeon's preference. Excess XP was suctioned away throughout the procedure. No mandatory soak or incubation time in the surgical cavity was required for XP's antimicrobial activity and therefore no increase in operating time was attributed to its use. Additionally, although XP was suctioned away, any residual solution remaining in the surgical cavity was not rinsed away due to its “no‐rinse needed” feature, enabling for its continued antimicrobial activity post closure.

XPERIENCE, an FDA cleared product was introduced to the US market in April 2021 for use as an intraoperative surgical irrigant to cleanse and remove debris including microorganisms, from the surgical wound space. It was designed based on a patented technology platform called XBIO® that utilizes material science approaches to prevent the formation of new biofilm, as well as treat and remove established biofilm. The XBIO technology incorporates a buffered pH modifier and surfactant, working synergistically to break down the biofilm matrix. By chelating metal ions from the EPS matrix, it facilitates the dissolution of polysaccharides and microbes, resulting in bacterial cell lysis within a high osmolarity environment. XPERIENCE was designed as a no‐rinse intraoperative surgical irrigation solution. It consists of citric acid, sodium citrate, and sodium lauryl sulfate in water. Citric acid and sodium citrate serve as pH buffers and aid in metal ion chelation, while sodium lauryl sulfate acts as a surfactant, assisting in debris removal including microorganisms.

### Data Collection

This retrospective study was conducted in accordance with ISO 14155 Standard for the Clinical Investigation of Medical Devices. The study protocol was reviewed and approved by the Hughston Foundation Sports Medicine Center Institutional Review Board (IRB) (#CSP‐021 v.3.0). Due to the retrospective and minimal risk nature of the study, a waiver of consent and authorization for the use/disclosure of protected health information was granted by the IRB. Only authorized surgical and research staff conducted an evaluation of the surgery schedule to identify the subjects who had undergone JAs between June 2021 and June 2022. Since XP administration at the time of surgery was not set as an identifiable parameter in the electronic medical systems, all records of subjects who had undergone primary knee, hip or shoulder arthroplasty were investigated. Once potential subjects were identified, all internal resources and databases housing medical records, charts, notes, and other relevant documentation were reviewed to further determine their suitability for inclusion in the study, based on defined inclusion/exclusion criteria listed below.

Inclusion criteria included: (i) the subject was 18 years of age or older; (ii) the subject had signed a surgical consent form, which did not prohibit use of protected health information; and (iii) the subject underwent a primary arthroplasty procedure between June 2021 and June 2022 and was administered XP intraoperatively.

Exclusion criteria included: (i) the subject was administered antibiotic beads during surgery; the subject did not have at least one follow‐up visit within 90 days after the index surgery; and (iii) the subject was from a vulnerable population, in accordance with 45 CFR 46 Subparts B, C, and D.

Once the inclusion/exclusion criteria were evaluated, relevant subject data (pursuant to waiver of HIPAA authorization criteria) was transferred to the Castor Electronic Data Capture (EDC) system, a cloud‐based clinical data management platform that operates in compliance with GCP regulations, and 21 CFR Part 11 of the U.S. FDA. The Castor EDC meets the International Organization for Standardization and International Electrotechnical Commission standards for designing, developing, providing, and maintaining an online platform for clinical research globally under ISO/IEC 27001:2013 and ISO 9001:2015. One hundred percent of the entries in Castor EDC were verified independently by two research staff and compared for accuracy. All diagnoses of PJI/PSI were re‐reviewed by the principal investigator following the CDC's National Healthcare Safety Network (NHSN) criteria for PJI^8^ while PSI diagnosis was determined by attending surgeons following the International Consensus Meeting (ICM) criteria.^10^

### Clinical Study Endpoints Evaluated

The primary end point evaluated for this retrospective analysis was the rate of PJI within 90 days of the index surgery following CDC's NHSN criteria. The additional endpoints evaluated were mean length of hospital stay and mean length of surgery. Collated data included: subject initials, date of birth, co‐morbidities, diagnosis, date of surgery, type of surgery, intraoperative use of antibiotics and irrigants in addition to XP, length of hospital stay, length of primary surgery and diagnoses of PJI within 90 days of index surgery.

### Statistical Evaluations

Statistical evaluations were performed in R programming environment and tables were constructed using gtsummary v1.7. Subject populations were separated according to hip, knee, or shoulder cohorts for analysis. Comparisons were made between surgical sites to investigate whether subject demographics differed per surgical site according to recorded metadata including age, sex, obesity (BMI > 30), race, diabetes, immunocompetence, chronic steroid use, heart disease, smoking, and alcohol use. XPERIENCE usage patterns were quantified according to how often it was used alone or with other infection prevention SOC regimens. Kruskal Wallis test was used to investigate whether operating time or length of hospital stay was non‐randomly distributed according to surgical site. Statistical approximations for PJI/PSI were made *via* the “rule of three” which states that if an event does not occur within *n* tries, then the true probability of that event lies between 0 and 3/n with a 95% confidence interval.

## Results

### Surgery Specifics and PJI/PSI Diagnosis

A total of 420 subjects underwent primary JAs at the hospital from June 2021 to June 2022 where XP was used intraoperatively. Three of those subjects underwent bilateral primary knee arthroplasties, and each bilateral surgery was included as separate primary index surgery resulting in a total of 423 separate index surgeries that were administered XP and evaluated in the study (217 knees, 164 hips and 42 shoulders) of which 97.16% of the procedures involved total joint arthroplasty surgeries. There were zero diagnoses of PJI and PSI noted in the 423 separate hip, knee, or shoulder arthroplasty surgeries within 90 days of the index surgery.

### Subject Demographics and Co‐morbidity Profiles

Table 1 lists the subject demographics in the total surgeries that were administered XP as well as based on the breakdown by the anatomical location of the surgery. There were a higher proportion of men and higher median age in the shoulder arthroplasty cohort compared to the hip and knee arthroplasty cohorts. Analysis of race characteristics determined a much higher proportion of white as opposed to other races in all arthroplasty cohorts.

**TABLE 1:** Subject demographics overall and in hip, knee, or shoulder arthroplasty procedures treated intraoperatively with XP.

Characteristic	Overall *N* = 423^†^	Hip *N* = 164^†^	Knee *N* = 217^†^	Shoulder *N* = 42^†^	*p*‐value*
Age	66 (59, 73)	64 (56, 71)	66 (60, 73)	72 (66, 76)	<0.001
Sex (female)	237 (56%)	95 (58%)	129 (59%)	13 (31%)	0.002
Race					
Black or African American	103 (24%)	44 (27%)	54 (25%)	5 (12%)	0.2
Other (Asian, American Indian, or Alaska Native	8 (1.9%)	2 (1.2%)	6 (2.8%)	0 (0%)	
White	312 (74%)	118 (72%)	157 (72%)	37 (88%)	

* Kruskal–Wallis rank sum test; Pearson's chi‐squared test; Fisher's exact test.

^†^ Median (IQR); *n* (%).

Table 2 lists the subject co‐morbidity profiles in the total surgeries that were administered XP as well as co‐morbidity profiles based on the breakdown by the anatomical location of the surgery. Overall, 403 (95%) of surgeries exhibited at least one of the listed co‐morbidity conditions with seven hips, 13 knees and 0 shoulders exhibiting none of the listed co‐morbidity conditions. The co‐morbidity conditions in descending order of prevalence were cardiovascular disease, obesity, current alcohol use, diabetes, and current nicotine use. Despite 95% of the subjects exhibiting at least one high risk condition, none of the subjects were diagnosed with early onset PJI within 90 days of surgery. Follow‐up of subjects for a minimum of 90 days to 15 months determined continued absence of early and delayed PJI/PSI diagnoses in the three cohorts.

**TABLE 2:** Subject co‐morbidity characteristics overall and in hip, knee, or shoulder arthroplasty procedures treated intraoperatively with XP.

Characteristic	Overall *N* = 423^†^	Hip *N* = 164^†^	Knee *N* = 217^†^	Shoulder *N* = 42^†^	*p*‐value*
BMI > 30	268 (63%)	101 (62%)	144 (66%)	23 (55%)	0.3
Diabetes	127 (30%)	43 (26%)	70 (32%)	14 (33%)	0.4
Immunocompromised	0 (0%)	0 (0%)	0 (0%)	0 (0%)	
Chronic steroid use	1 (0.2%)	1 (0.6%)	0 (0%)	0 (0%)	0.5
Cardiovascular disease	324 (77%)	128 (78%)	161 (74%)	35 (83%)	0.4
Current nicotine use	32 (7.6%)	16 (9.8%)	10 (4.6%)	6 (14%)	0.027
Current alcohol use	153 (36%)	62 (38%)	76 (35%)	15 (36%)	0.9

* Kruskal–Wallis rank sum test; Pearson's chi‐squared test; Fisher's exact test.

^†^ Median (IQR); *n* (%).

### Intraoperative Surgical Irrigation/Antimicrobial Regimens

Table 3 lists the intraoperative use of XP as either the sole intervention or its use in combination with other antiseptic and antimicrobial regimens in accordance with personalized procedures implemented by the operating orthopedic surgeon. The use of XP as the sole antimicrobial intraoperative intervention was noted to be higher in the hip and knee cohorts compared to the shoulder cohort. In surgeries that included the intraoperative use of an antibiotic powder, vancomycin was the preferred intraoperative antibiotic regimen compared to tobramycin. In surgeries that included the intraoperative use of an antimicrobial/antiseptic irrigant in addition to XP, Betadine® was used in the vast majority of the surgeries with hydrogen peroxide and Clorpactin used in one surgery each.

**TABLE 3:** Intraoperative XP intervention with or without additional antimicrobial interventions.

	Anatomical site			
	Hip	Knee	Shoulder	Total
Treatment				
XP	73 (17%)	70 (17%)	4 (0.9%)	147 (35%)
XP + Antibiotic powder (vancomycin or tobramycin)	8 (1.9%)	19 (4.5%)	5 (1.2%)	32 (7.6%)
XP + other irrigant (Betadine and/or hydrogen peroxide and/or Clorpactin)	78 (18%)	95 (22%)	7 (1.7%)	180 (43%)
XP + Antibiotic powder + other Irrigant	5 (1.2%)	33 (7.8%)	26 (6.1%)	64 (15%)
Total	164 (39%)	217 (51%)	42 (9.9%)	423 (100%)

### Operating Time and Length of Stay

Table 4 lists the mean total operating times in hours and mean length of stay in days for the surgeries overall as well as based on the breakdown by the anatomical location of the surgery. The shoulder arthroplasty cohort exhibited the lowest mean operating times and mean length of stay, while the hip cohort exhibited the highest mean operating times and mean length of stay. In all cohorts the mean operating time was less than 90 min. With the absence of PJI/PSI diagnosis, the evaluation of correlation of operating times and length of stay with infection diagnoses was not germane.

**TABLE 4:** Mean operating times and length of stay overall and in hip, knee, or shoulder arthroplasty procedures treated intraoperatively with XP.

Characteristic	Overall *N* = 423^†^	Hip *N* = 164^†^	Knee *N* = 217^†^	Shoulder *N* = 42^†^	*p*‐value*
Total operating time (hours)	1.31 (0.34)	1.45 (0.34)	1.24 (0.32)	1.10 (0.21)	<0.001
Length of stay (days)	1.07 (0.87)	1.26 (0.86)	0.96 (0.91)	0.83 (0.49)	<0.001

* Kruskal–Wallis rank sum test.

^†^ Mean (SD).

## Discussion

The principal finding in this retrospective study was the absence of acute PJI diagnoses in 423 JAs that had received XP as the surgical irrigant either solely or in combination with other surgical irrigation and antimicrobial infection prevention regimens.

### Prevalence and Projections of Total JAs

The number of elective total JAs in the USA is expected to rise significantly, with predicted total annual counts for primary THA in the United States for 2025, estimated at 652,000 and primary TKA predicted total annual counts for 2025 estimated at 1,272,000.^2^ In contrast, shoulder arthroplasty volumes are predicted to increase, to 350,558 procedures by 2025 outpacing the growth rate projections of THA and TKA.^3^ In recent years (2018–2021), all three surgical procedures, THA, TKA and TSA have been removed from the Centers for Medicare and Medicaid Services in‐patient only lists contributing to the increase in the number of joint replacement surgeries being performed.

### Periprosthetic Joint Infections and Biofilm

Periprosthetic joint infection is a devastating and costly complication following total JA. The incidence of PJI ranges from 1% to 2% in primary THA and TKA procedures but can be higher^4^, ^11^, ^12^, ^13^, ^14^ while incidence rates of PSI in TSA can range from 0.4% to 4%.^15^, ^16^ Eighty percent of infections in humans and potentially PJIs, can be attributed to a microbial biofilm matrix.^17^, ^18^, ^19^, ^20^, ^21^, ^22^, ^23^ Bacteria protected within the biofilm matrix are far more resistant to antibiotics, antimicrobials, antiseptics, and host immune defenses making their removal notoriously difficult when matured.^24^, ^25^ Thus, preventing the formation of recalcitrant biofilm matrix is critical to an infection prevention strategy. The utilization of intraoperative irrigation solutions to decrease contaminating bioburden in the surgical wound remains an important strategy.^6^, ^26^, ^27^, ^28^, ^29^

### Rationale for Evaluating XPERIENCE as an Intraoperative Surgical Irrigant

Although intraoperative surgical irrigation remains an important infection prevention component, there is no accepted standard of care. The FDA cleared intraoperative surgical irrigant XP, has been determined to be biocompatible and safe. The biocompatible and minimally cytotoxic nature of the product allows for the “no‐rinse” feature of XP, which lends to its ease of use during the surgical irrigation procedure and potentially enables residual XP in the surgical wound space to continue its prophylactic activity for an extended period after surgical incision closure.^30^, ^31^ In *in vitro* studies, XP exhibited antimicrobial activity against all bacteria evaluated, including planktonic and superstructure biofilms formed from microorganisms like *Pseudomonas aeruginosa*, *Staphylococcus aureus* and *Cutibacterium acnes* determined to be causative agents of PJIs/PSIs.^31^, ^32^ Additionally, XP exhibited equivalent, or better antimicrobial activity against microorganisms in comparison to Betadine solution (10% w/v Povidone Iodine [PI]).^31^, ^32^ Dilute PI has been evaluated for use by the CDC and World Health Organization as an intraoperative irrigant.^33^, ^34^ In contrast to XP, dilute PI when used intraoperatively in joint replacement surgeries requires a soak time followed by an extensive rinse with saline solution.^35^ In *in vitro* phenotypic and functional evaluations using an osteoblast cell line, PI negatively impacted cell line viability and functionality in a statistically significant manner when compared to XP.^36^ In a prospective clinical study, irrigation with XP during primary TKA was noted to decrease post‐operative swelling and improve limb functionality in a statistically significant manner when compared to irrigation with dilute PI.^37^ Thus, XP could have perceived advantages of ease of use, safety and multi‐functional efficacy compared to PI.

### Clinical Endpoints Evaluated in the JA Cohorts

This retrospective study evaluated the impact of XP on PJI and PSI in 423 separate primary hip, knee and shoulder joint arthroplasties performed in a hospital setting. The personalized use of XP by multiple surgeons during their JAs either as the sole antimicrobial regimen or in combination with antibiotic powders and other antiseptic/antimicrobial irrigants, represented its real‐world utilization in surgical settings. The foremost findings were that there were zero early onset PJI/PSI diagnosed within 90 days of index surgery and continued absence of delayed PJIs following all three cohorts for at least 90 days and up to 15 months for the earliest surgeries. Demographic evaluation of the cohorts determined that 95% of the subjects presented with at least one or more high risk, co‐morbidity profile(s) that would have rendered a predisposition to PJI.^38^ Despite the high risk, co‐morbidity profiles of the cohorts, there were no early onset or delayed PJI/PSI diagnosed in this retrospective study. Evaluation of operating times indicated that mean operating time lasted 1.31 h considering all surgeries. Although the mean total operating time for the complete JA cohort was less than 90 min, several of the surgeries had operating times of greater than 90 min. In literature reports, operative time of greater than 90 min in primary JA had a significantly higher incidence of PJI (1.4%) compared to cases lasting less than 90 min (0.7%).^39^ The statistical rule of three would result in a maximum true infection rate of 0.70% (3/423) based on zero events in 423 attempts. This would indicate maximum PJI/PSI rates in this retrospective study at or below the historical PJI/PSI rate. In instances of THA following failed osteosynthesis, a much higher PJI rate of greater than 6% has been noted.^40^ Although this retrospective study was limited to primary total JA cohorts, it would be relevant to evaluate efficacy of irrigation with XP either solely or in combination with other intraoperative infection prevention strategies to decrease PJI rates in THA cohorts following failed osteosynthesis. This retrospective study potentially positions XP as a promising intraoperative, no‐rinse required, antimicrobial irrigant that can be easily incorporated into a broader primary PJI/PSI prevention strategy.

### Limitations

This retrospective analysis included a small cohort of 423 primary hip, knee and shoulder arthroplasty surgeries treated intraoperatively with XP either on its own or with additional intraoperative surgeon preferred antimicrobial and antiseptic regimens. The study lacked inclusion of a comparable control cohort of primary hip, knee and shoulder arthroplasty surgeries that were not treated with XP. Given that the surgeries were performed by at least nine different surgeons, each with their personalized SOC and infection prevention regimens, a definitive finding of the efficacy of XP in preventing PJI/PSI in this retrospective clinical study will need to be validated in well‐controlled prospective clinical studies comparing the sole use of XP intervention with other intraoperative infection prevention modalities to evaluate its comparability or superiority to other intraoperative antimicrobial irrigants currently in use. Although the data was collated from several detailed sources, the original purpose of the source data information was not intended for research purposes and therefore would not be appropriate for nuanced evaluation of other parameters that could indicate benefits or detriments of XP in addition to its efficacy on PJI and PSI determined in the study.

## Conclusions

This study evaluated the efficacy of the novel XP in preventing PJI/PSI in 423 separate hip, knee, and shoulder index arthroplasties. Surgeries were conducted at a hospital in the USA and were performed by at least nine orthopedic surgeons, each using their preferred perioperative SOC regimens, preferred surgical approach techniques, and preferred infection prevention procedures. The intraoperative use of XP as an antimicrobial irrigant by the various surgeons possibly varied. This study is a true representation of the real‐world use of XP and could reflect its efficacy in decreasing PJI/PSI incidence in the field. In this retrospective clinical study, XP appeared to be efficacious at preventing PJI at rates at or below historical reported incidences of PJI. This study warrants further investigations of XP as the sole antimicrobial irrigant in robustly designed, controlled, blinded, prospective multi‐center clinical trials in JA surgeries to determine potential utility of this novel irrigant to lessen the overall burden of PJI and improve patient outcomes, of which some clinical studies are in progress.^41^

## Conflict of Interest Statement

The authors have no conflicts of interest to declare.

## Ethics Statement

The study protocol was reviewed and approved by the Hughston Foundation Sports Medicine Center Institutional Review Board (IRB), and it conforms to the provisions of the Declaration of Helsinki (as revised in Brazil in 2013). Waiver of informed consent: According to 45 CFR 46.116(f)(3), an IRB may waive the requirements to obtain informed consent provided that the IRB finds and documents that: the research involves no more than minimal risk to the subjects, the waiver will not adversely affect the rights and welfare of the subjects, the research could not practicably be carried out without the waiver, and whenever appropriate, the subjects will be provided with additional pertinent information after participation.

## Author Contributions

All authors had full access to the data in the study and bear responsibility for the integrity of the data and the accuracy of the data analysis. Conceptualization, R.H.; methodology, R.H. and M.W.; data curation, M.W.; investigation, R.H. and M.W; formal analysis, R.H. and M.W.; resources, R.H.; writing—original draft, R.H. and M.W.; writing – review and editing, R.H. and M.W.; funding acquisition, R.H.

## Funding Information

The clinical study received funding for IRB review and Castor EDC platform from Hughston Foundation.
